# New Operation upon the Shaft of Long Bones, by Which Elongation as Well as Straightening, May Be Secured

**Published:** 1867-12

**Authors:** J. C. Hughes

**Affiliations:** The Iowa State Medical Society


### NEW OPERATION

### *Upon the Shaft of Long Bones, by which Elongation, as well as Straightening, may be Secured.*

REPORTED BY PROF. J. C. HUGHES, M. D., TO THE IOWA STATE MEDICAL SOCIETY.

The subject of deformities connected with the different parts of the body, has been receiving much attention from the profession within the last quarter of a century, and many valuable improvements for their correction have been suggested. But perhaps none has received less attention than those arising from fracture, when, from either maltreatment, or a want of the healthy process of nutrition and deposit, these malpositions have been induced. As a curative means, in some of these deformities, Professor Barton, of Philadelphia, has done much to warrant success; and his mode of removing a V shaped portion of bone from the convexity of the curve, is all that would seem to be demanded where straightening alone of the extremity is sought for.

Patients, as well as Surgeons, are often satisfied with this result, thinking it all that science or art can accomplish.

The case here reported, suggested to my mind, some years ago, a new mode of treatment. In order to accomplish a cure, it was not only necessary that the limb should be straightened, but length must be secured as well. Barton's treatment would not accomplish this. Could anything more be done?

James H. Dimond, aged 13, during the month of October, 1856, was thrown from a horse-cart, producing a comminuted fracture of the left femur, in its upper third. The limb was immediately placed in position, and dressed with the long splint—*Desault modified by Physic.* No unpleasant symptoms attended the treatment; and at the expiration of eight weeks, the splints were removed. Assured of the necessity of further protection to the limb, I continued dressings to the leg for twenty days longer, when I removed the entire dressings, the limb giving evidence of perfect recovery. He now began to move about upon crutches, and in a few days was engaged in the sports upon the snow with a hand-sled. At the expiration of two months—which time I had been absent from the city—the patient presented himself at my office, with the leg, to my great astonishment, so distorted and shortened, that, upon measurement, I found a difference of two and one-quarter inches — the convexity of the bend being just below the great trochanter, forming a very angular curve. Upon enquiry, I found the bending to have been gradual, having commenced soon after the removal of the dressings, but now so firm as to forbid any hope of restoration from treatment, short of operative interference. The breaking down of new deposit, by the establishment of fracture, in a case which had been comminuted, did not offer much in the way of cure. The deformity, however, continued to increase, until the shortening measured two and three-fourth inches, interfering materially with locomotion.

During the Summer of 1857, he became anxious to have the limb straightened, and consulted me several times. I gave him some encouragement in relation to the result which might follow an operation, but advised him to wait until the Fall. I frequently examined it after that time, and, weighing the chances of a cure, by *Barton's* method of operating, concluded to suggest what I conceived to be an improvement — one in which, if the operation could be made successful, would not only correct the deformity produced by the curve, but would restore the limb to its natural length. After having secured the consent of patient and friends, I presented the claims of the operation to my colleagues in the Medical Department of the Iowa University, who thought favorably of the new method suggested, and sanctioned its adoption. On the 5th of December, 1857, before the Medical Class of the University I presented the patient, and after a full explanation of the case — the dangers, as well as what I conceived to be the advantages of the operation — I proceeded in the following manner. So determined was the patient to have the operation performed, that when upon the operating table, just before commencing the administration of chloroform, he used the following significant language: "*Here goes for a straight leg or a dead body!*"

Having prepared a needle six inches in length, of malleable iron, I attached to it the chain saw; then, with the scalpel, made an incision some three inches in length, directly over the greatest convexity of the curve, and laid bare the bone, which was, at this point, almost, if not entirely, twice its natural thickness. I now entered the needle on the inside, and, with the use of considerable force, pushed it around, hugging the bone so closely with the needle's point, as not to endanger vessels or nerves, and doing but little injury to the soft parts. As the point of the needle reached the opening or entrance on the opposite side — having made the circumference of the bone,—I took hold of its point with a strong pair of forceps, and forced it through, leaving the chain-saw in contact with at least three-fourths of the circumference of the bone, including the concavity of the curve. Having secured for the saw the position desired, I at once affixed the handles, and proceeded to cut through from the inner or concave surface. Not wishing to sever the entire thickness of the femur, I made the incision extend about three-fourths through, and then, with pressure from the outside, or from the convex surface, broke down the outer fourth, which, with the periostium and soft tissues, kept up the attachments between the extremities of the bone. Having now removed the saw, I was enabled to make extension which would give me the full length of the sound limb. The gap or space secured, by which I gained length, was on the inner or concave surface, instead of the outer or convex, as in Barton's method. I had not removed a wedge shaped portion of bone, as by the old method; but had secured, by making my incision from the inside, a wedge-shaped space, which, when extension was produced, could fill up and be united by bony deposit.

I at once placed the limb in *Desault's* long splint, and treated as for compound fracture. The danger to be anticipated from the injury inflicted upon the tissues, and the presence of bone dust produced by the saw, would be active inflammation, followed by extensive suppuration. This was, however, more imaginary than real. The case progressed most favorably, scarcely maintaining the ordinary symptoms of compound fracture. But little suppuration followed—the discharge of pus being confined to the original wound. In ten weeks and three days he was removed to his home, one mile from the hospital; and in four months from the operation, he was able to attend school. His health improved, the leg became strong; and in a few months I found him acting as bell-boy in one of our hotels, making good time either up or down a staircase. The limb, which had been two and three-fourth inches shortened, was now within half an inch of the length of the sound one. At the outbreak of the war, he applied for admission to the ranks, but his age prevented. Determined to serve his country in some capacity, he became mess boy to the officers, and discharged his duty faithfully until the latter part of 1862, or early in the year 1863, when he again offered himself as a volunteer—was accepted, and mustered into the 30th Iowa Infantry. He was prompt and faithful as a soldier, always at his post, and in that memorable charge on the 22d of May, 1863, at Vicksburg, he was among the number of brave boys who paid the forfeit with their lives. May his name live with his country, and his courage and bravery mark an epoch in surgical science.

